# Variation Patterns of Functional Trait Moments Along Geographical Gradients and Their Environmental Determinants in the Subtropical Evergreen Broadleaved Forests

**DOI:** 10.3389/fpls.2021.686965

**Published:** 2021-07-12

**Authors:** Caishuang Huang, Yue Xu, Runguo Zang

**Affiliations:** ^1^Research Institute of Forest Ecology, Environment and Protection, Chinese Academy of Forestry, Key Laboratory of Forest Ecology and Environment of National Forestry and Grassland Administration, Beijing, China; ^2^Co-Innovation Center for Sustainable Forestry in Southern China, Nanjing Forestry University, Nanjing, China

**Keywords:** biogeography, biodiversity conservation, climate variability, environmental factors, functional diversity, trait moment

## Abstract

Understanding how environmental change alters the composition of plant assemblages is a major challenge in the face of global climate change. Researches accounting for site-specific trait values within forest communities help bridge plant economics theory and functional biogeography to better evaluate and predict relationships between environment and ecosystem functioning. Here, by measuring six functional traits (specific leaf area, leaf dry matter content, leaf nitrogen, and phosphorus concentration, leaf nitrogen/phosphorus, wood density) for 292 woody plant species (48,680 individuals) from 250 established permanent forest dynamics plots in five locations across the subtropical evergreen broadleaved forests (SEBLF) in China, we quantified functional compositions of communities by calculating four trait moments, i.e., community-weighted mean, variance, skewness, and kurtosis. The geographical (latitudinal, longitudinal, and elevational) patterns of functional trait moments and their environmental drivers were examined. Results showed that functional trait moments shifted significantly along the geographical gradients, and trait moments varied in different ways across different gradients. Plants generally showed coordinated trait shifts toward more conservative growth strategies (lower specific leaf area, leaf N and P concentration while higher leaf nitrogen/phosphorus and wood density) along increasing latitude and longitude. However, trends opposite to the latitudinal and longitudinal patterns appeared in trait mean values along elevation. The three sets of environmental variables (climate, soil and topography) explained 35.0–69.0%, 21.0–56.0%, 14.0–31.0%, and 16.0–30.0% of the variations in mean, variance, skewness, and kurtosis across the six functional traits, respectively. Patterns of shifts in functional trait moments along geographical gradients in the subtropical region were mainly determined by the joint effects of climatic and edaphic conditions. Climate regimes, especially climate variability, were the strongest driving force, followed by soil nutrients, while topography played the least role. Moreover, the relationship of variance, skewness and kurtosis with climate and their geographical patterns suggested that rare phenotypes at edges of trait space were selected in harsher environments. Our study suggested that environmental filtering (especially climate variability) was the dominant process of functional assembly for forest communities in the subtropical region along geographical gradients.

## Introduction

Functional traits are defined as any measurable phenological, morphological, physiological, or regenerative characteristics at the individual plant level (Violle et al., 2014), which directly or indirectly affect species performance (i.e., metabolism, adaptation, resource utilization strategy) and ecosystem functioning (Liu et al., 2019). Knowledge on plant functional traits plays an important role in improving our understanding of community assembly and ecosystem functioning (McGill et al., 2006). To date, the studies of a single functional trait have been widely conducted in many ecosystem types (Yu et al., 2018; Liu et al., 2019). However, there is evidence that studies based on only a single or few functional traits may limit the predictive power of the models of ecosystem responses (Savage et al., 2007). Instead, a suite of functional traits or the combination of functional traits such as the Leaf-Height-Seed strategy framework, which was considered to better reflect important functional axes that drive plant performance (Golodets et al., 2010; Wieczynski et al., 2019), are necessary to model ecosystem response to environmental change.

Functional diversity measures the range, abundance and distribution of functional traits, which has been widely used in biodiversity and ecosystem functioning research (Enquist et al., 2015). Given the increasingly important role of functional diversity in community ecology, biogeography, and conservation biology, there is an urgent need to develop studies on functional diversity and associated metrics (Violle et al., 2017). Functional trait moments can describe the distribution of functional traits in communities and reflect the functional composition and abundance of ecological strategies displayed by co-occurring species (Enquist et al., 2015). The first four trait moments (i.e., mean, variance, skewness, and kurtosis) capture the key features of functional distribution by characterizing the dominance, dispersion, rarity, and evenness of functional trait values within communities (Enquist et al., 2015), which are important for explaining the variation in multiple ecosystem functions, tracking the biodiversity loss and carrying out conservation planning (Bagousse-Pinguet et al., 2021). Particularly, weighting trait moments according to individual abundance can accurately quantify the shapes of whole-community trait distributions and more strongly relate to overall ecosystem function (Newbold et al., 2012; Gross et al., 2018).

The biogeography of plant function is a foundation of plant ecology. Taking the functional traits into account in biogeographical research is likely to provide insights into predicting the biogeographical patterns of ecosystem functioning and to decipher which environmental variables affect different aspects of ecosystem functioning across space (Swenson et al., 2011; Liu et al., 2019). With the development of functional biogeography, there are more and more studies on the geographical patterns of functional diversity. For instance, most studies at forest communities revealed that the functional composition decreased from lower latitudes/elevations to higher latitudes/elevations due to the decreased temperature (Apaza-Quevedo et al., 2015; Wieczynski et al., 2019). However, previous studies on the biogeographical variations of plant functional traits considered only latitudinal or elevational gradients, few studies have been done to test variations of functional diversity along multiple gradients simultaneously. Although the latitudinal gradients and elevational gradients are often considered to be parallel in many studies (Chun and Lee, 2018), increased rather than decreased trends of functional diversity with elevation were found in other studies on low or middle altitude mountains (Zhang et al., 2017). Therefore, it is still necessary to study the variations of functional diversity along different geographical gradients.

Geographical variation of plant functional traits is closely related to the current environment. The environmental filtering hypothesis proposed that the abiotic factors acted as “filters” to select species to adapt to local conditions from the regional pool according to sets of functional traits (Le Bagousse-Pinguet et al., 2017). Regional environmental factor (e.g., climate) was demonstrated as the most important factor in shaping the geographical pattern of dominant trait values at a large scale (Zhou et al., 2013; Shiono et al., 2015), while local conditions (soil and topography) more strongly affect the trait dissimilarity (Bello et al., 2013). Many previous studies showed that climate predominantly determined functional diversity. For instance, seasonal climate could affect the growth length of plants and is considered to be a key factor reducing the variance and range of functional traits (Swenson et al., 2012). Wieczynski et al. (2019) found that climate governs functional diversity at a global scale by using an approach based on trait moments. In addition, local conditions are non-negligible. Ecologists found that much of the large variation in functional traits occurring in any given climate might be explained by local conditions (Reich and Oleksyn, 2004). Therefore, understanding the relative effects of regional and local environmental factors on functional diversity remains an important challenge. Our study considered regional and local factors simultaneously in their effects on plant functional diversity, which may contribute to the accurate prediction of functional trait–environment relationships in a changing future (Shipley et al., 2016).

China is a country with the largest and most typical subtropical evergreen broadleaved forest (SEBLF) in the world. Affected by Tibetan plateau, SEBLF of China is under the subtropical monsoon climate featured by distinct four seasons (Tang, 2010). The SEBLF is characterized with complex floristic components, rich biodiversity, and high endemism (Zhou et al., 2013). Although progress has been made in trait-environment relationships in SEBLF communities (Shiono et al., 2015), few studies have simultaneously combined the abundance-weighted trait moments with a range of environmental variables using location-specific trait measurements.

Here, we focused on SEBLF in China, using a method based on abundance-weighted trait moments to assess how environmental conditions influence the trait composition of forest communities. We used several plant functional traits (specific leaf area, leaf dry matter content, wood density, leaf nitrogen concentration, phosphorus concentration, and leaf nitrogen/phosphorus ratio) and sets of environmental variables (climate, topography, and soil) to explore the functional variations of forest communities along geographical and environmental gradients. We hypothesized that: (i) The trait moments (i.e., mean, variance, skewness, and kurtosis) show significant spatial patterns and vary in similar ways with different geographical gradients. (ii) Climate primarily drives the geographical variations of the four trait moments. (iii) Stressful and variable environment can reduce the variance of functional traits and increase the kurtosis by selecting species that are functionally similar within communities. We hope this study can provide an insightful understanding of the importance of functional diversity and its relationships with abiotic conditions in functional biogeography.

## Materials and Methods

### Study Area

We randomly established 50 permanent forest dynamics plots (FDPs) with an area of 20 m × 20 m in each site across five provinces (Zhejiang province, Jiangxi province, Anhui province, Chongqing municipality, and Sichuan province) in the subtropical region of China. In total, 250 FDPs were established in natural old-growth evergreen broadleaved forests (27.58°–30.18°N, 102.95°–120.00°E), with the elevation ranging from 200 to 1948 m. All FDPs were established and investigated according to the standard of the Center for Tropical Forest Science (CTFS) (Condit, 1998) during the summer of 2018 and 2019. For woody plant species, all individuals with the diameter at breast height ≥ 1 cm were tagged, mapped, and identified to species level with the help of local botanists. The abundances of species were determined by calculating the number of individuals of a certain species in each plot. In total, 292 woody plant species (48,680 individuals) belonging to 59 families and 134 genera were collected.

### Trait Data

Woody plants can optimize their growth and survival across environmental gradients by investing energy differentially in leaf and wood tissues (Fortunel et al., 2014). In this study, six functional traits regarding leaf and wood trait axes were measured in 2018 and 2019: specific leaf area (SLA, cm^2^ g^–1^), wood density (WD, g cm^–3^), leaf dry matter content (LDMC, g g^–1^), leaf nitrogen concentration (LNC, g kg^–1^), leaf phosphorus concentration (LPC, g kg^–1^), and leaf nitrogen/phosphorus ratio (N/P, %). These key functional traits are believed to influence plant performance and reflect life history strategies in plants. In particular, SLA is related to net photosynthetic rate and relative growth rate (Hulshof et al., 2013). LDMC is related to nutrient retention within the plant (Ryser and Urbas, 2003). LNC and LPC affect photosynthetic rate and stomatal conductance (Chaturvedi et al., 2012). N/P is used to determine whether plant growth is restricted by nutrients (Gusewell, 2004). WD is related to the efficiency and safety of water transport (Cornwell et al., 2006), affecting species recruitment in particular sites (Zanne et al., 2018). Integrating these leaf and wood trait axes to define functional strategies for woody plants provides an estimation of ‘whole-plant’ response to environmental changes (Fortunel et al., 2012).

In each 20 m × 20 m plot, ten individuals of every species were sampled. For those species with less than ten individuals, we added additional individuals of the same species from surrounding areas. For each individual, we selected five intact, fully expanded leaves for measuring the leaf-related functional traits. For each leaf, the fresh leaf area was measured using a scanner and the area was calculated using image analysis software ImageJ^1^. Leaf fresh weight was measured in the field. Dry weight was obtained after oven-drying at 80°C for 48 h. We calculated SLA as the ratio of fresh leaf area to dry mass. LDMC was calculated as the ratio of oven-dry mass to water-saturated fresh mass. LNC and LPC were determined by Kjeldahl digestion, followed by colorimetric analysis (Perez-Harguindeguy et al., 2013). N/P was the ratio of leaf nitrogen concentration to leaf phosphorus concentration. To avoid adverse effects on tree growth, wood samples were taken from 1 to 2 cm diameter branches instead of tree cores. These wood samples were oven-dried for 72 h at 105°C after removal of the bark. WD was calculated as the ratio of the dry mass to their fresh volume, measured using the water displacement method with Mettler-Toledo balance.

### Trait Moments Calculation

Among the four trait moments, community-weighted mean represents the dominant functional trait values in a community (Lajoie and Vellend, 2018), which can strongly determine ecosystem functioning (Violle et al., 2017). Community-weighted variance is a measure of dispersion of functional trait values within communities, which can provide an accurate measure of the volume of niche space (Gaedke and Klauschies, 2017). Community-weighted skewness describes the symmetry of functional trait distributions. Higher absolute values indicate the existence of a few abundant species with extreme trait values typically resulted from asymmetric competition or rapid environmental change (Enquist et al., 2015). Community-weighted kurtosis depicts the peaking of functional trait distributions, where high values indicating a high abundance of species with similar trait values thus low trait diversity, which might be caused by environmental filtering. By contrast, low values reflect an even abundance distribution of trait values and a high trait diversity possibly resulted from limiting similarity (Cornwell and Ackerly, 2009; Katabuchi et al., 2012). We used the following equations to calculate abundance-weighted functional trait moments within each plot (Wieczynski et al., 2019):

$$Community-weightedmean=CWM=\frac{\sum w_{i}x_{i}}{\sum w_{i}}$$

$$C\text{om}munity-weighted\;v\text{a}riance=CWV=\frac{\sum w_{i}{\left(x_{i}-CWM\right)}^{2}}{\sum w_{i}}$$

$$C\text{omm}unity-weighted\;s\text{ke}wness=CWS={\frac{\sum w_{i}\left(\frac{x_{i}-CWM}{\sqrt{CWV}}\right)}{\sum w_{i}}}^{3}$$

$$C\text{omm}unity-weighted\mathrm{kurt}osis=CWK={\frac{\sum w_{i}\left(\frac{(x_{i}-CWM}{\sqrt{CWV}}\right)}{\sum w_{i}}}^{4}-3$$

Where w_*i*_ is the abundance of the ith species in each plot and x_*i*_ is the specific mean trait values of the ith species.

Since extreme outliers (e.g., values of kurtosis > 100) would completely bias the results, outliers were removed by dropping the outer 1% (0.5% highest and 0.5% lowest) of species mean and community moment values for each functional trait (Wieczynski et al., 2019).

### Environmental Variables

To estimate the influences of environmental variables on community-level trait moments, we collected climatic, edaphic, and topographic variables to represent environmental conditions. 19 bioclimatic variables for each plot were gathered using climate raster layers with a high-resolution (30 arcsec) available online^2^.

At the central point and four corners of each plot, five soil samples were collected from 0 to 20 cm depth. We mixed the five soil samples thoroughly into one bulked sample and air-dried it to constant weight. The samples were tested for soil pH, soil organic matter, total nitrogen, total phosphorus, available nitrogen, available phosphorus, and soil available potassium. Two topographic variables were also measured at the center of each plot, including slope and aspect. The slope (Slo) is the mean angle of inclination of the four triangular planes composed of any three quadrat corners. Aspect (Asp) was the values from 0° to 360° measured in degrees from north, indicating the azimuth.

There were high pairwise correlations among these environmental variables. In order to avoid collinearity, Pearson’s correlation values (*R* < 0.8) between these variables were used as a cutoff criterion to retain variables that were more relevant to the response variables. We retained four climatic variables, including mean diurnal range (MDR), mean temperature of wettest quarter (MTWQ), precipitation seasonality (PS), and precipitation of warmest quarter (PWQ), three edaphic variables including soil total nitrogen (STN), soil total phosphorus (STP), and soil available potassium (SAK), and two topographic variables including slope (Slo) and aspect (Asp) ultimately. The correlations between environmental variables were shown in supporting information (Supplementary Table 1).

### Data Analysis

We first used linear regressions to test shifts in trait moments along altitude, longitude, and elevation. We then analyzed the pairwise relationships between trait moments and environmental variables by using Pearson correlation. Beforehand, we examined the spatial autocorrelation of all functional trait moments by calculating global Moran’s *I*. Significant positive spatial autocorrelation was revealed for all trait moments (Moran’s *I* values ranged from 0.115 to 0.653, *P* < 0.01), resulting in more significant results when examining the significance of models (Lennon, 2000). Since spatial autocorrelation produces a bias in the estimation of correlation coefficients, a modified *t*-test approach was used to eliminate the spatial autocorrelation (Dutilleul, 1993; Sadoti et al., 2010). The modified *t*-test creates an effective sample size that takes into account the spatial structure by introducing the estimated covariance matrixes for the distance classes and then corrects the correlation coefficient between the trait moments and environmental variables (Dutilleul, 1993). Finally, we calculated the mean magnitudes (absolute values) of all corrected correlation coefficients to detect the environmental variables most correlated with trait moments. Moreover, we conducted variance partitioning to analyze the relative contributions of environmental factors in determining functional diversity. This approach segregates total variation in the community matrix (i.e., trait moments) into climatic, edaphic, and topographic components with corresponding *P*-values by using a partial regression method (Legendre and Legendre, 1998).

We also conducted a principal component analysis (PCA) on the combined trait moments of all functional traits and identified the primary drivers of multidimensional trait variation to compare with each functional trait individually (Wieczynski et al., 2019). A redundancy analysis (RDA) was also performed to explore how much variance in different trait moments could be explained by environmental variables. Both PCA and RDA analyses revealed similar results, so we provided the confirmatory information in the supporting information (Supplementary Figures 1, 2).

In order to fit assumptions about the uniformity of data and the homoscedasticity of errors, trait data were log-transformed prior to analysis. All of the statistical analyses were performed in R 3.4.3 (R Development Core Team 2017). Linear regressions were calculated with the R package ‘lm’. Modified t-tests were calculated with the ‘spatialpack’ package. Partial regressions were conducted with the ‘vegan’ package.

## Results

### Shifts in Trait Moments Along the Geographical Gradients

**FIGURE 1:**
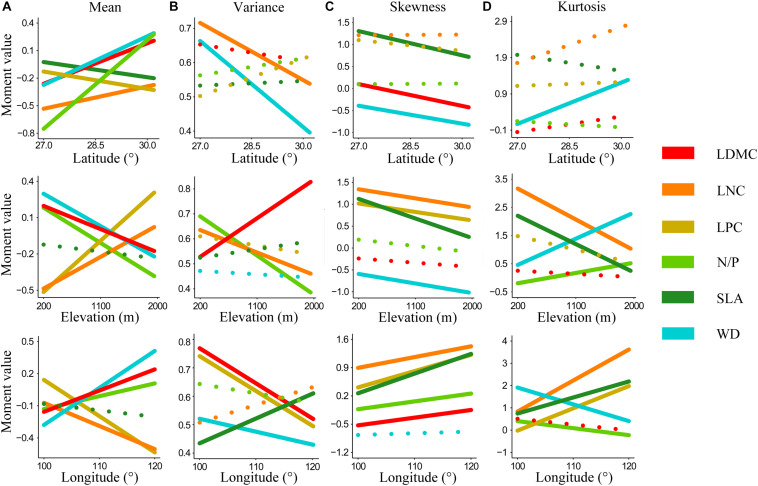
Shifts in the community-weighted moments of six key plant functional traits along geographical gradients. **(A–D)** Abundance-weighted moments of whole-community functional trait distribution across latitude, elevation, and longitude. Lines represent linear regressions on trait moment values and geographical gradients. Solid lines signify significant regressions. Functional traits are abbreviated as follows: leaf dry matter content (LDMC), leaf nitrogen concentration (LNC), leaf phosphorus concentration (LPC), leaf nitrogen/phosphorus ratio (N/P), specific leaf area (SLA), wood density (WD).

Trait moments shifted significantly along geographical gradients (Figure 1). And different trait moments varied in different ways across geographical gradients. As latitude and longitude increased, mean values of N/P, LDMC, and WD increased, while mean of SLA and LPC decreased significantly. Opposite tendencies appeared in these functional traits along elevation, with mean of N/P, LDMC, and WD decreased significantly yet mean of LPC increased (Figure 1A). Most traits in variance decreased with increasing gradients, although the significantly decreased traits varied in different gradients (i.e., WD and LNC for latitude, LNC and N/P for elevation, LDMC, LPC, and WD for longitude) (Figure 1B). In addition, skewness of most functional traits increased along longitude yet decreased along both latitude and elevation (except skewness of LNC and N/P, which increased slightly along latitude) (Figure 1C). For kurtosis, only WD increased significantly with increasing latitude. Kurtosis of most functional traits decreased along elevation, while kurtosis of WD and N/P increased significantly. With the increase of longitude, kurtosis of WD and LDMC decreased significantly while kurtosis of SLA, LNC, and LPC increased significantly (Figure 1D).

### Correlations of Functional Trait Moments With Environmental Variables

**FIGURE 2:**
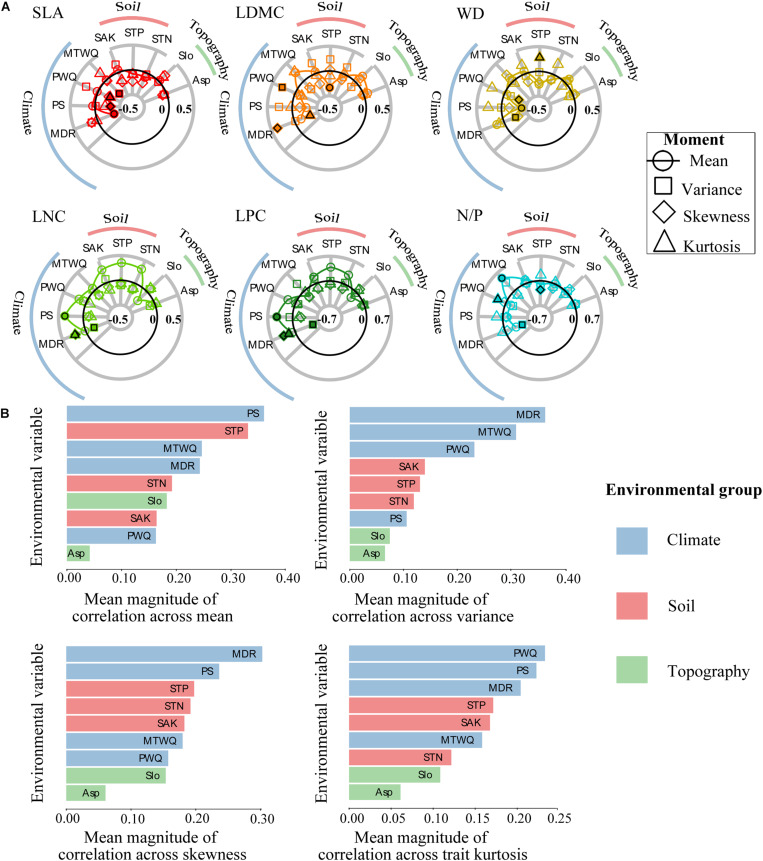
Relationships between the community-weighted moments of individual functional traits and the environmental variables across all forest plots. **(A)** Radar plots showing all trait moment–environment correlations. Each symbol represents a different trait moment, and its position along the radial axis indicates the strength of correlation between trait moment and a given environmental variable. The solid black line represented zero correlation, the region inside (outside) this line represents negative (positive) correlations. Filled shapes highlight the environmental variables that are most strongly correlated with each of the four trait moments. **(B)** Histograms showing mean magnitudes (absolute values) of all correlations between all trait moments and each environmental variable. Environmental variables are abbreviated as follows: mean temperature of wettest quarter (MTWQ), mean diurnal range (MDR), precipitation of warmest quarter (PWQ), precipitation seasonality (PS), soil total nitrogen (STN), soil total phosphorus (STP), soil available potassium (SAK), slope (Slo), aspect (Asp). Functional traits are abbreviated as follows: leaf dry matter content (LDMC), leaf nitrogen concentration (LNC), leaf phosphorus concentration (LPC), leaf nitrogen/phosphorus ratio (N/P), specific leaf area (SLA), wood density (WD).

Strong functional trait moment–environment relationships were found in the SEBLF (Figure 2 and Supplementary Figure 1). Climate exhibited the strongest correlations on average across all trait moments, while topography had the least correlation with the four trait moments (Figure 2B). More specifically, precipitation seasonality (PS) and soil total phosphorus (STP) were the strongest predictors of trait mean. Mean diurnal range (MDR) and mean temperature of wettest quarter (MTWQ) were the strongest predictors of variance. MDR and PS were the strongest predictors of skewness. Precipitation of warmest quarter (PWQ) and PS were the strongest predictors of kurtosis. Our pairwise analysis (Figure 2) identified the same environmental correlates as identified via alignment with trait_PC1 and trait_PC2 in Supplementary Figure 1. There were striking similarities in the correlations between environmental variables and functional trait composition (Figure 2A). For instance, the mean values of traits linked with resource conservation (LDMC, WD, and N/P) was negatively related to precipitation (PS, PWQ) and soil nutrient measures [STP and soil total nitrogen (STN)], while positively related to temperature (MTWQ). Meanwhile, mean values of traits related to resource acquisition (LNC and LPC) were positively related to climate (PS, PWQ, MDR) and soil [STN, STP, soil available potassium (SAK)]. The variance of WD, LPC, and N/P was negatively correlated to MDR and PWQ while positively correlated to MTWQ. The skewness of SLA, WD, LPC, and N/P was positively correlated to MDR, while negatively related to PS, MTWQ, and STP. Interestingly, trait variance and kurtosis had opposite relationships with environmental variables in 70% (37 of 54) of the cases.

### Variance Partitioning of Trait Moments for Environmental Variables

**FIGURE 3:**
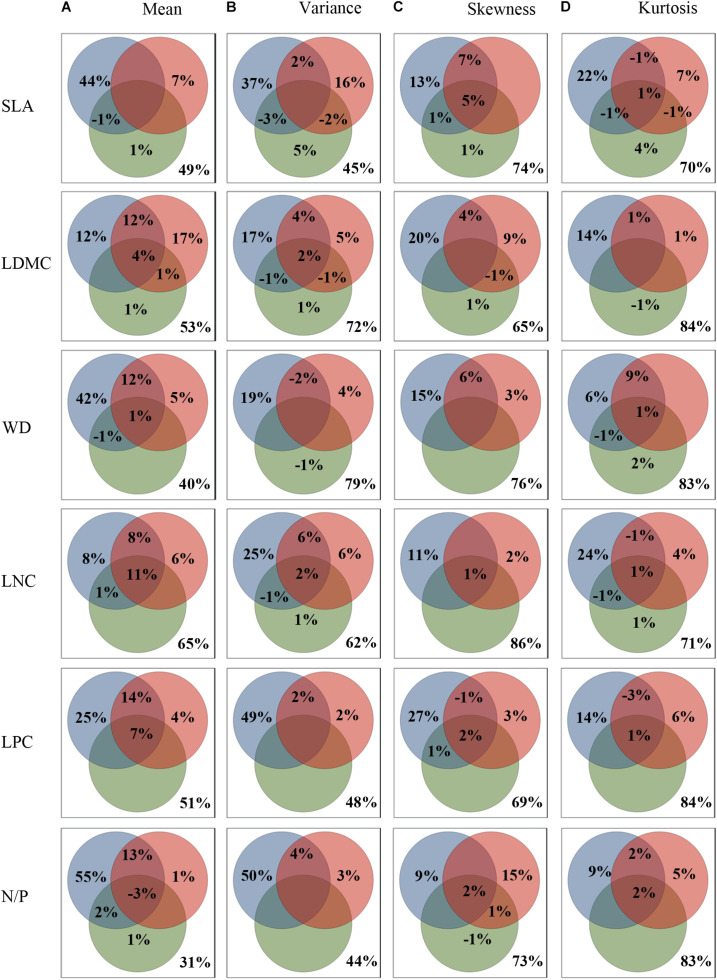
Variance partitioning of community-weighted trait moments across all functional traits explained by environment. **(A–D)** showing the variance partitioning of climatic (blue), edaphic (red), and topography (green) variables in accounting for the variance in community-weighted trait moments. Functional traits are abbreviated as follows: leaf dry matter content (LDMC), leaf nitrogen concentration (LNC), leaf phosphorus concentration (LPC), leaf nitrogen/phosphorus ratio (N/P), specific leaf area (SLA), wood density (WD).

Results of the variance partitioning analysis were shown in Figure 3. The three sets of environmental variables explained 35.0–69.0%, 21.0–56.0%, 14.0–31.0%, 16.0–30.0% of the variations in mean, variance, skewness and kurtosis across all functional traits, respectively. And the results were consistent with the results of RDA analysis (Supplementary Figure 2). Obviously, the explanatory power of environmental variables for CWMs was much higher than the others. The variance of functional trait moments explained by the three sets of explanatory variables showed similar patterns (Figures 3A–D), namely, climate (9.0–55.0%) played a dominant role in determining the functional trait patterns of the SEBLF in China, followed by soil (0.0–17.0%) and finally topography (0.0–5.0%). The regional climate and local conditions (soil and topography) jointly affected the geographical variation of trait moments, especially for trait mean.

## Discussion

The distribution of trait values often deviates from normal distribution. Investigating trait distributions offers a unique opportunity to understand the interplay of functional dominance, variation, rarity, and evenness (Bagousse-Pinguet et al., 2021). Trait distribution may change in response to environmental selection and indicate the prevailing selection pressure (Gaedke and Klauschies, 2017). Therefore, it is necessary to describe the shape of trait distributions systematically, as well as to discover and quantify the relationships between environment and trait moments (Swenson and Weiser, 2010).

Trait moments can reflect the strategies of plants to adapt to environmental changes along different geographical gradients (Kang et al., 2011). Across the study areas, substantial shifts in trait moments along gradients of latitude, longitude, and elevation were found. However, in contrast with our first hypothesis, trait moments varied in different ways across geographical gradients. Opposite tendencies appeared in these functional traits along latitude and elevation in particular. Plants generally showed coordinated trait shifts toward more conservative (slow) growth strategies to adapt to the adverse environment along latitude and longitude (Reich, 2014; Wieczynski et al., 2019). Species with lower SLA and LPC, higher LDMC and WD (e.g., *Camellia fraterna, Eurya rubiginosa var. attenuata*) were dominant at higher latitudes and longitudes. Nevertheless, notable examples of trends opposite to the above-mentioned latitudinal patterns appeared in trait mean along elevation—higher LNC and LPC, lower LDMC and WD. The opposite trends indicated that latitude and elevation were not completely interchangeable proxies for measuring changes in trait composition in response to environment across space (Hulshof et al., 2013; Wieczynski et al., 2019). Generally, variance and skewness decreased, while kurtosis increased with these geographical gradients. These trends suggested that rare phenotypes at edges of trait space were selected in harsher environments. However, these trait moments of different functional traits also exhibited divergent trends across these gradients, such as variance and kurtosis of LDMC, LPC, N/P, and skewness of N/P, LNC. These divergences suggested that environmental conditions of different geographical gradients might have different effects on different trait axes (Wieczynski et al., 2019).

Given that geographical patterns of trait composition were closely linked to environmental factors (Liu et al., 2019), these divergences could be explained by relationships between individual environmental variables and geographical gradients (Wieczynski et al., 2019). The nine environmental variables showed distinct geographical patterns in our study area. In particular, some environmental variables (such as MDR, PS, MTWQ, and STP) that were strongly associated with trait moments exhibited different correlations with these gradients (Supplementary Table 1). For instance, soil total phosphorus was strongly positively correlated with elevation while negatively correlated with longitude, thus mean values of leaf phosphorus concentration increased along increasing elevation yet decreased along increasing longitude (Figure 1). Therefore, these results indicated that more detailed analyses about the relationships between individual environmental variables and functional composition are necessary.

Quantifying relationships between environment and functional composition is necessary to predict responses of community and ecosystem to future environmental change (Funk et al., 2017). This work provided strong evidence that the four trait moments are crucial metrics for improving our understanding of the effects of environmental changes on plant communities and ecosystem functions (Enquist et al., 2015; Le Bagousse-Pinguet et al., 2017). Our study showed that 35.0–69.0% of the total variation in trait mean was explained by the three sets of environmental variables (Figure 3A), followed by variance (21.0–56.0%; Figure 3B). By contrast, the variation explained for the skewness (14.0–31.0%; Figure 3C) and kurtosis (16.0–30.0%; Figure 3D) was relatively lower. The highest percentage of variations in these trait moments were accounted for by climate (Figure 3), which supported our second hypothesis that climate was a key driving factor for the functional diversity of SEBLF in China. In particular, climate variability (MDR and PS) exhibited the strongest correlations across all trait moments. It was consistent with a previous study conducted in monsoon climate regions of Japan (Shiono et al., 2015). The woody plant assemblages in the study region have developed under a warm and wet monsoon climate, where the extreme climate may not be able to create a strong effect on the persistence of many species. In contrast, climatic factors related to climate variability can alter the length of growing season and phenology for plants and play a more important role in shaping the geographical patterns of functional community structures (Figure 2 and Supplementary Figures 1, 2; Zhou et al., 2013).

Together, regional climate and local soil represented important factors shaping the functional composition of highly diverse subtropical forests, highlighting climate variability as potentially the primary drivers of this variation (Figures 2, 3 and Supplementary Figures 1, 2). Precipitation seasonality was strongly negatively correlated with the mean of wood density and leaf dry matter content, while positively correlated with the mean of leaf nutrient concentrations (Figures 2, 3). It suggested that seasonal precipitation may favor species with efficient water transport and nutrient utilization to support fast growth during the wet season (Liu et al., 2020). Results also showed that soil total nitrogen and total phosphorus were strongly positively correlated with those mean of functional traits representing soil resource use efficiencies (leaf nitrogen concentration and phosphorus concentration). These results together indicated that functional trait assembly of woody plant assemblage is to a large extent sorted along the gradients of climate variability (Shiono et al., 2015). Community functional composition shifted according to changes in environmental factors: larger precipitation fluctuation and more soil fertility shift community functional composition toward species with smaller leaf and wood tissue densities, and thus faster growth through greater resource acquisition. It is also noteworthy that the effect of soil on trait moment patterns was mostly nested within the effect of the climate, especially on trait mean (Figure 3). Climate might be both a major control of functional traits and an important driver of soil development (Reich and Oleksyn, 2004), leading to an interactive effect of soil and climate on the geographical patterns of plant functional traits. For instance, recent studies have found that greater precipitation seasonality can accelerate soil organic matter decomposition and increase soil nutrients, which might be a reason for the strong positive correlations between PS and leaf nutrient traits in this study (Ma et al., 2015).

Importantly, there was strong evidence that variance of five of the six functional traits (LDMC, WD, LNC, LPC, and N/P) was found to be negatively correlated with mean diurnal range (stressful and variable climate), while kurtosis of SLA, WD, LNC, LPC, and N/P was positively correlated with mean diurnal range. It was in accordance with our third hypothesis, which predicted that stressful and variable environment can result in a reduction in the variance of functional trait values and increase the kurtosis by selecting species that are functionally similar within communities (Simova et al., 2015; Le Bagousse-Pinguet et al., 2017). In addition, we showed that the trait variance and kurtosis were negatively correlated and coordinated along the environmental gradients (Figure 2 and Supplementary Table 2), associating with different trait assembly processes (Wieczynski et al., 2019). For instance, we found lower trait variance (negative correlation) while higher kurtosis (positive correlation) in WD, LNC, LPC, and N/P along the gradient of unstable environment (MDR), which suggested that environmental filtering results in forest communities in which functionally similar species tend to co-occur. These results were consistent with a recent theory which suggests that rapid fluctuations in temperature should cause whole-community trait distributions to track the changing environment by decreasing fitness and growth of dominant phenotypes while increasing fitness and growth of some currently rare phenotypes (Enquist et al., 2015; Gross et al., 2018; Wieczynski et al., 2019). Therefore, our study suggested that temperature fluctuation probably acts as a stressor limiting functional diversity and fitness in SEBLF. However, we showed only limited evidence that precipitation seasonality restricted variance of functional traits. We even found even (low kurtosis) and broad (high variance) distributions of SLA, LNC, LPC, and LDMC along the gradient of precipitation seasonality. Our results suggested that PS did not necessarily limit functional diversity (Swenson et al., 2012), but rather permits various functional strategies to cross the filtering effect imposed by an abiotic stress to co-occur within assemblages in the region (Le Bagousse-Pinguet et al., 2017). Likewise, negative correlations between PS and skewness in most functional traits indicated that some currently adapted phenotypes with suits of functional traits become increasingly dominated under seasonal precipitation. Accordingly, we inferred that PS probably acts as a driver leading to trait divergence of woody plants and developing optimum functional strategies in SEBLF. Our results are consistent with previous studies showing that climate variability acts not only as a filter of species traits but also as a driver creating a greater difference in functional strategies among woody plant species (Shiono et al., 2015). Examining multiple metrics and environmental variables simultaneously can not only reveal the response of different aspects of functional diversity to environmental changes, but also provide a profound understanding of the community assembly process.

Studies found that climate change poses a significant impact on biodiversity. Rapid changes in temperature or precipitation will affect the trait distribution. If the rate of change exceeds the pace of biological response, especially the capacity of communities to track and adapt to climate change, impacts on community function and structure may be profound (Ackerly et al., 2010). For instance, when temperature fluctuation occurs, the fitness and growth of existing phenotypes within communities will shift over time, with mean and skewness deviating from the initial distribution in directions determined by their underlying relationships with temperature, thus affecting ecosystem function (Wieczynski et al., 2019). Apparently, approaches based on functional trait moments can capture key features of trait distribution, potentially improve the accuracy of trait-climate relationships, and help to select appropriate metrics for the research of biodiversity conservation under the current climate change (Giovani et al., 2018). A range of conservation measures, including expanded reserve systems, intensive management, and the more controversial idea of managed translocation, are urgently needed to reduce the impacts of climate change on biodiversity and ecosystem services.

The strongest trait-environment linkages were achieved when using community-weighted mean, suggesting that environment may structure large-scale community composition by selecting for certain optimal functional trait values (Simova et al., 2015). However, as mentioned above, the predictive power of environmental factors was decreased when explained the higher-order moments of functional trait distributions. Intraspecific functional trait variability is often considered contributing to a considerable amount of trait variation (Violle et al., 2012; Enquist et al., 2015). However, only mean species functional trait values were used in our study, which may lead to unreliable measures of the shape of functional trait distributions. Another possible explanation is that some important factors were missed in our study, such as dispersal limitations or biotic interactions (Schamp et al., 2008; Gross et al., 2009). For instance, dispersal barriers may act as a stochastic factor that hinders the effect of climate on the functional trait structure by influencing the species assemblage patterns (Shiono et al., 2015). Asymmetry competition between individuals might result in skew distributions of functional trait values (Ding et al., 2019). Competition between functionally similar species could increase the functional evenness (Katabuchi et al., 2012). The expectation is that incorporating these factors in future research would increase the predictive power of the trait-environment relationships we found.

## Conclusion

Understanding the functional diversity variations and their determinants of the SEBLF, a typical forest ecosystem type in subtropical region, is necessary for biodiversity conservation or vegetation restoration. Our study is, to our knowledge, the first attempt to investigate the functional diversity patterns by combining the four abundance-weighted functional trait moments with regional and local environmental drivers simultaneously in the SEBLF of China. Our study found significant variations of functional diversity along three geographical gradients, reflecting directional shifts in ecological strategies of plants in response to changing environmental conditions. Climate regimes (especially climate variability as the major driving factor) and local conditions jointly influenced the geographical patterns of functional trait composition in the subtropical forest communities. Temperature fluctuation might act as a filter of limiting functional diversity, while seasonal precipitation probably acted as a driver for forest communities to develop optimum functional strategies in utilizing and competing for local resources in subtropical regions. This research provided a more comprehensive and detailed understanding of the complex role of the environment on the functional biogeography of forest communities in the subtropical region. The results about relationships between functional trait moments and climatic factors may provide valuable insights for biodiversity conservation under changing climate regimes.

## Data Availability Statement

The original contributions presented in the study are included in the article/Supplementary Material, further inquiries can be directed to the corresponding author/s.

## Author Contributions

RZ and YX conceived this project. CH and YX conducted the field investigation and collected the data. CH performed the statistical analyses and wrote the first draft with YX. All the authors contributed to improving the quality of the manuscript.

## Conflict of Interest

The authors declare that the research was conducted in the absence of any commercial or financial relationships that could be construed as a potential conflict of interest.

## Footnotes

1 http://rsbweb.nih.gov/ij/

2 https://www.worldclim.org/
