# Supplementary material for: Variation Patterns of Functional Trait Moments Along Geographical Gradients and Their Environmental Determinants in the Subtropical Evergreen Broadleaved Forests
Source: Front Plant Sci. 2021 Jul 12;12:686965. doi: 10.3389/fpls.2021.686965 (PMC8311185; doi:10.3389/fpls.2021.686965)
Supplement: Supplementary file 1 [file Data_Sheet_1.docx]

**Supplementary materials**

**Tables**

**Table S1** Correlations between environmental variables. Significant levels:

*, p < 0.05; **, p < 0.01; ***, p < 0.001.

|  | Lon | Lat | Elev | Asp | Slo | STN |
| --- | --- | --- | --- | --- | --- | --- |
| Asp | -0.09^**^ | 0.11^***^ | 0.10^**^ |  |  |  |
| Slo | 0.47^***^ | -0.33^***^ | -0.47^***^ | -0.08^*^ |  |  |
| STN | -0.70^***^ | 0.42^***^ | 0.78^***^ | 0.08^*^ | -0.50^***^ |  |
| STP | -0.72^***^ | 0.22^***^ | 0.74^***^ | -0.03 | -0.49^***^ | 0.78^***^ |
| SAK | -0.33^***^ | 0.53^***^ | 0.40^***^ | -0.04 | -0.27^***^ | 0.71^***^ |
| MTWQ | -0.13^***^ | 0.45^***^ | -0.23^***^ | 0.12^***^ | -0.12^***^ | 0.09^**^ |
| PWQ | -0.33^***^ | 0.31^***^ | 0.55^***^ | -0.03 | -0.25^***^ | 0.45^***^ |
| PS | -0.93^***^ | 0.26^***^ | 0.91^***^ | 0.08^**^ | -0.52^***^ | 0.76^***^ |
| MDR | 0.21^***^ | -0.03 | 0.23^***^ | -0.03 | -0.04 | 0.07^*^ |

|  | STP | SAK | MTWQ | PWQ | PS |
| --- | --- | --- | --- | --- | --- |
| SAK | 0.59^***^ |  |  |  |  |
| MTWQ | -0.04 | 0.07^*^ |  |  |  |
| PWQ | 0.47^***^ | 0.49^***^ | -0.51^***^ |  |  |
| PS | 0.77^***^ | 0.47^***^ | -0.04 | 0.61^***^ |  |
| MDR | 0.16^***^ | 0.00 | -0.66^***^ | 0.36^***^ | 0.01 |

Environmental variables are abbreviated as follows: mean temperature of wettest quarter (MTWQ), mean diurnal range (MDR), precipitation of warmest quarter (PWQ), precipitation seasonality (PS), soil total nitrogen (STN), soil total phosphorus (STP), soil available potassium (SAK), slope (Slo), aspect (Asp).

**Table S2** Correlations between variance and kurtosis of functional traits. Significant levels:

*, p < 0.05; **, p < 0.01; ***, p < 0.001

| Variance  Kurtosis | WD | SLA | N/P | LPC | LNC | LDMC |
| --- | --- | --- | --- | --- | --- | --- |
|  |  |  |  |  |  |  |
| WD | -0.18^**^ | -0.1 | 0.01 | 0.03 | -0.14^*^ | -0.15^*^ |
| SLA | -0.21^***^ | -0.59^***^ | -0.12 | -0.27^***^ | -0.46^***^ | -0.35^***^ |
| N/P | -0.02 | 0.1 | -0.36^***^ | -0.18^**^ | 0.05 | 0.06 |
| LPC | -0.25^***^ | -0.28^***^ | -0.37^***^ | -0.47^***^ | -0.38^***^ | -0.31^***^ |
| LNC | -0.33^***^ | -0.29^***^ | -0.26^***^ | -0.42^***^ | -0.47^***^ | -0.32^***^ |
| LDMC | -0.07 | -0.2^**^ | 0.23^***^ | 0.14^*^ | -0.08 | -0.42^***^ |

Functional traits are abbreviated as follows: wood density (WD), leaf dry matter content (LDMC), specific leaf area (SLA), leaf nitrogen/phosphorus ratio (N/P), leaf nitrogen concentration (LNC), leaf phosphorus concentration (LPC).

**Figures**


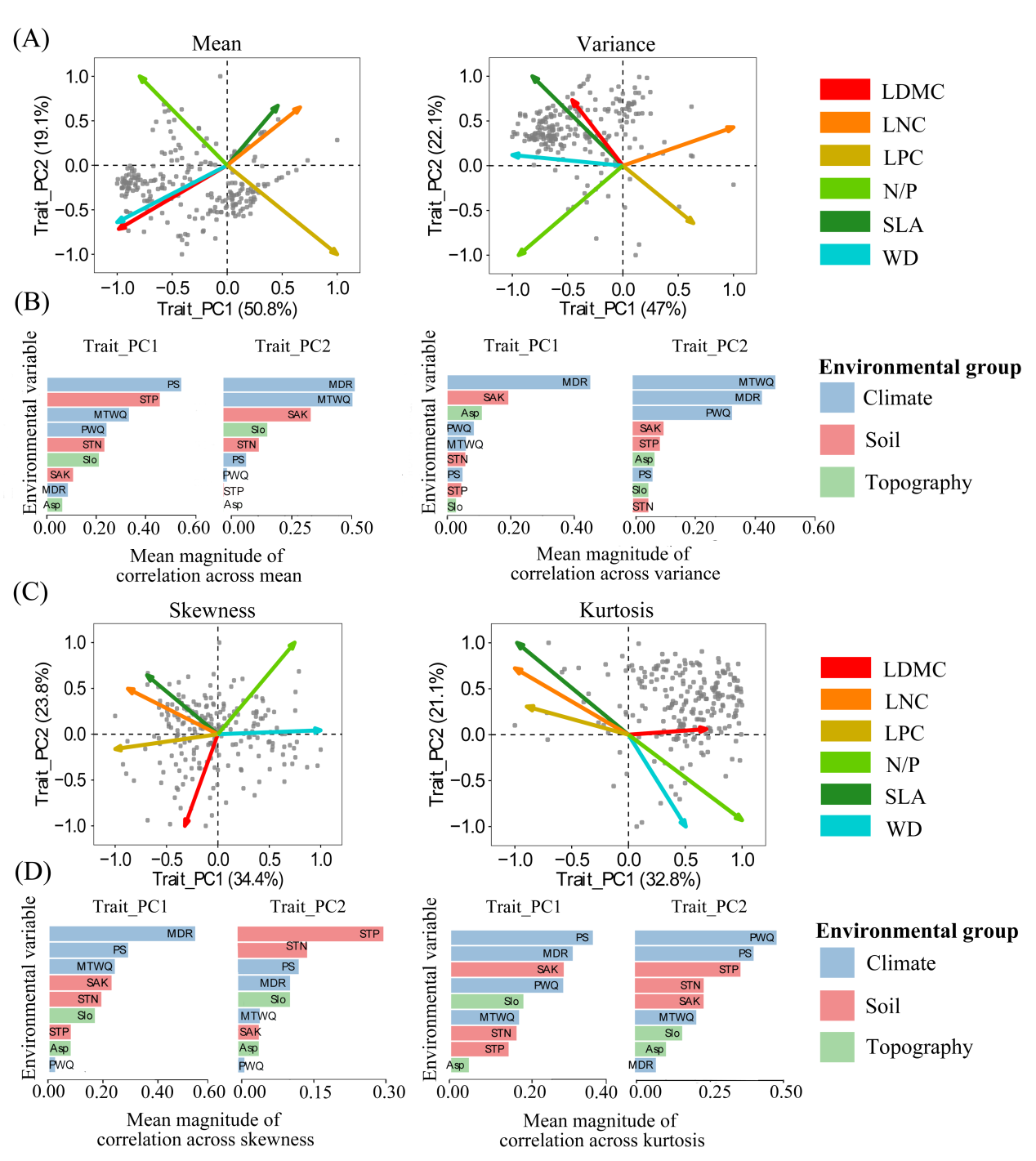


**Figure S1** The first two principal component axes of community-weighted trait moment (Trait_PC1 and Trait_PC2) across all functional traits and communities and the strength of their correlations with each environmental variable. (A and C) The first two principal trait axes. Gray points show the principal coordinates for each community, and colored lines represent loadings (eigenvalues) for individual functional traits. Both the coordinates and loadings have been rescaled to the interval [−1,1]. (B and D) Correlations between the first two principal trait axes and individual environmental variables. Environmental variables are abbreviated as follows: mean temperature of wettest quarter (MTWQ), mean diurnal range (MDR), precipitation of warmest quarter (PWQ), precipitation seasonality (PS), soil total nitrogen (STN), soil total phosphorus (STP), soil available potassium (SAK), slope (Slo), aspect (Asp). Functional traits are abbreviated as follows: wood density (WD), leaf dry matter content (LDMC), specific leaf area (SLA), leaf nitrogen/phosphorus ratio (N/P), leaf nitrogen concentration (LNC), leaf phosphorus concentration (LPC).

**
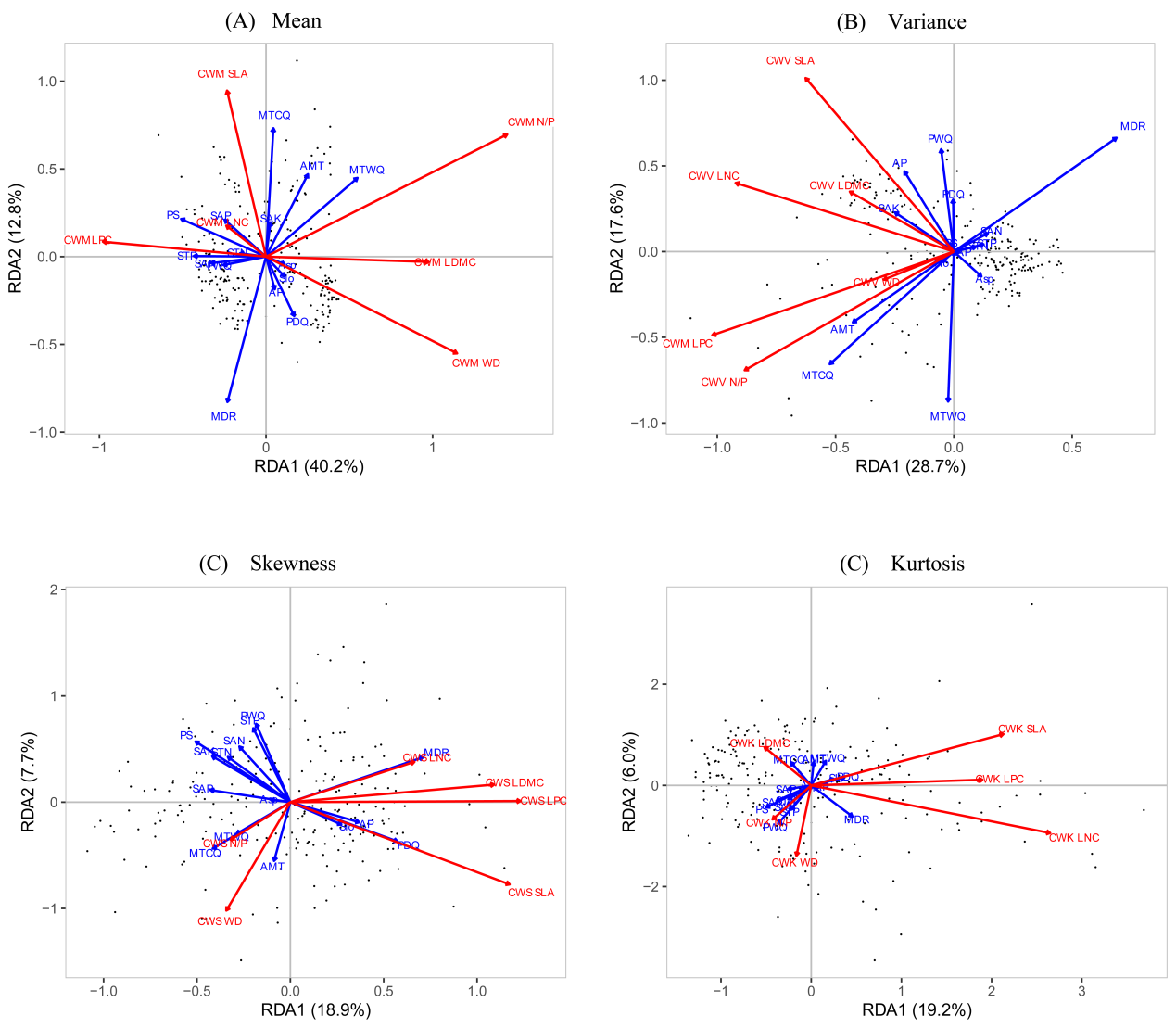
**

**Figure S2** Redundancy analysis (RDA) ordination biplot of individual functional trait moments and environmental variables. The blue arrows represent environmental factors and the red arrows represent trait moments. Environmental variables are abbreviated as follows: Aspect (Asp), Slope (Slo), soil total nitrogen (STN), soil total phosphorus (STP), soil available potassium (SAK), soil available nitrogen (SAN), soil available phosphorus (SAP), annual mean temperature (AMT), mean temperature of wettest quarter (MTWQ), mean temperature of coldest quarter (MTCQ), annual precipitation (AP), precipitation of warmest quarter (PWQ), precipitation of driest quarter (PDQ), precipitation seasonality (PS), mean diurnal range (MDR). Functional traits are abbreviated as follows: wood density (WD), leaf dry matter content (LDMC), specific leaf area (SLA), leaf nitrogen/phosphorus ratio (N/P), leaf nitrogen concentration (LNC), leaf phosphorus concentration (LPC).
